# Different Tactics of Synthesized Zinc Oxide Nanoparticles, Homeostasis Ions, and Phytohormones as Regulators and Adaptatively Parameters to Alleviate the Adverse Effects of Salinity Stress on Plants

**DOI:** 10.3390/life13010073

**Published:** 2022-12-27

**Authors:** Mostafa Ahmed, Kincső Decsi, Zoltán Tóth

**Affiliations:** 1 Festetics Doctoral School, Institute of Agronomy, Georgikon Campus, Hungarian University of Agriculture and Life Sciences, 8360 Keszthely, Hungary; 2 Department of Agricultural Biochemistry, Faculty of Agriculture, Cairo University, Giza 12613, Egypt; 3 Institute of Agronomy, Georgikon Campus, Hungarian University of Agriculture and Life Sciences, 8360 Keszthely, Hungary

**Keywords:** abiotic stress, salinity, zinc oxide nanoparticles, phytohormones, ion homeostasis

## Abstract

A major abiotic barrier to crop yield and profitability is salt stress, which is most prevalent in arid and semi-arid locations worldwide. Salinity tolerance is complicated and multifaceted, including a variety of mechanisms, and to adapt to salt stress, plants have constructed a network of biological and molecular processes. An expanding field of agricultural research that combines physiological measures with molecular techniques has sought to better understand how plants deploy tolerance to salinity at various levels. As the first line of defense against oxidative damage brought on by salt stress, host plants synthesize and accumulate several osmoprotectants. They (osmoprotectants) and other phytohormones were shown to serve a variety of protective roles for salt stress tolerance. Intrinsic root growth inhibition, which could be a protection mechanism under salty conditions, may be dependent on phytohormone-mediated salt signaling pathways. This article may also make it easier for scientists to determine the precise molecular processes underlying the ZnO-NPs-based salinity tolerance response for some plants. ZnO-NPs are considered to improve plant growth and photosynthetic rates while also positively regulating salt tolerance. When plants are under osmotic stress, their administration to zinc nanoparticles may also affect the activity of antioxidant enzymes. So, ZnO-NPs could be a promising method, side by side with the released osmoprotectants and phytohormones, to relieve salt stress in plants.

## 1. Introduction

Salt-affected soils are those that have an excessive amount of soluble salts or exchangeable sodium in the root zone. In dry and semi-arid regions of the world, crop production is now seriously threatened by salt stress because of poor rains, high runoff demand, and ineffective soil and water management techniques [1]. No climate zone is exempt from the danger of salinization, despite the widespread belief that it mainly affects arid and semi-arid areas [2]. According to the FAO handbook for saline soil management that was released in 2018, salinity has had a detrimental effect on 242 million hectares, and sodification (increasing the percentage of sodium in the irrigation water) may harmfully affect millions of others—especially as the previous percent just monitored in Eurasian countries. The previous numbers mean that millions of global arable land hectares are in a dangerous situation [3].

Plants can generally be divided into two major types; one is called glycophytes and another group is halophytes. This classification depends on the climatic conditions they can survive in cases of highly salinized environments. Halophytes are plants that can withstand salinities comparable to those found in the saltwater of the sea or, in some circumstances, can tolerate even higher levels [4]. Limiting salt intake, lowering the concentration of salt inside the cytoplasm and cell wall through accumulating large amounts of Na^+^ in the vacuoles, and ion compartmentation are the three primary strategies followed by halophytes to combat the negative consequences of high salt concentrations. The two techniques used by halophytes to alleviate the bad impacts of excessive salts are ineffective for glycophytes or other salt-sensitive plants that cannot withstand high salinity levels. As a result, they boost hazardous amounts of salt in the cytosol [5].

**Figure 1:**
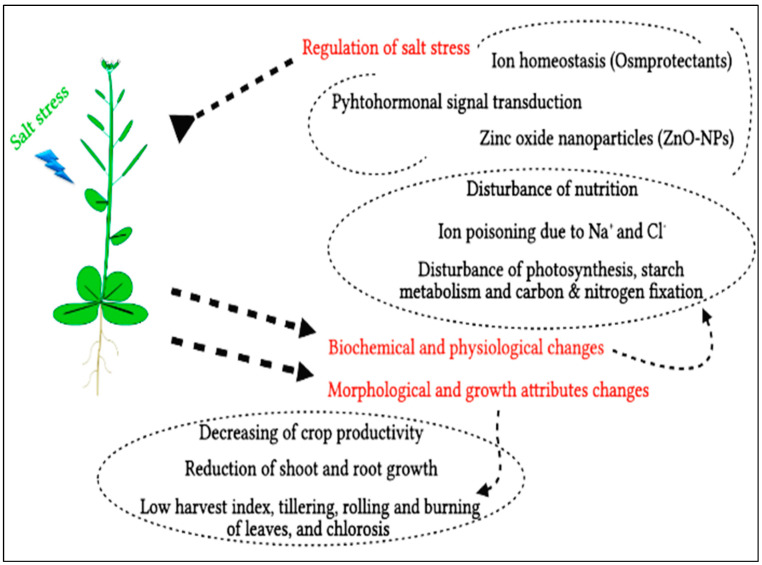
The harmful effects of salt stress on plants and different methods of regulation.

Salt stress can cause many problems on different levels, such as molecular, morphological, and biochemical (metabolic). For these harmful effects, there are many types of regulation parameters that are responsible for maintaining the negative effects of that kind of abiotic stress in plants, as shown in Figure 1. There are three possible influences of salt stress on crop yield; high osmotic stress because of the low supply of extrinsic water, ion poisoning due to sodium and/or chloride, or disturbed nutrition as a result of some interferences in the uptake and transport of some crucial nutritional elements [6]. The Osmo-stress is associated with ion accretion in the solutions of the soil, while nutritional disturbance and particular ion impacts are linked to ion accumulation—especially Na^+^ and Cl^−^ that may be found in dangerous levels that affect the accessibility of other crucial elements, such as Ca and K [7]. When bio-membranes and substructural organelles in plant organs are damaged by toxic quantities of sodium, growth is inhibited and aberrant depression occurs before plant fatality [8]. Under saline circumstances, other physiological systems, including photosynthesis, starch anabolism, respiratory processes, and nitrogen-fixing, are also disrupted, resulting in decreases in crop yield.

Many years ago, the textile, agricultural, and pharmaceutical industries saw a significant increase in the demand for transdisciplinary applications of developed nanoparticles (NPs) [9,10,11]. Growers and profit-seeking enterprises are particularly interested in using NPs in the field of agriculture as insecticides, fertilizers, or regulators in crop growth and development. By eliminating or reducing the pathogenic attack, NPs may improve food outputs [11]. In contrast to traditional chemical substances—which are regularly subject to penetration, steaming, photo-hydrolytic compensation, and microbial degeneration—these nanoproducts allow small amounts of fertilizers, insecticides, or pesticides to be used efficiently over a specific time, while their design allows for steady release and resistance to acute environmental harshness [12].

Recently, green, biogenic, or chemically-synthesized nanoparticles have received significant attention from a lot of researchers across many fields, such as medicine and agriculture, as these newly developed nanoparticles are feasible, less toxic, have a wide and permanent effect, and are cost-effective. The synthesized nanoparticles are utilized considerably to protect plant growth and development against variant kinds of abiotic stresses, such as salinity, chilling, drought, flooding, toxic heavy metals, excessive temperatures, etc. [13]. Accordingly, it was noted that zinc oxide nanoparticles (ZnO-NPs) play vital and crucial roles in plant growth and improvement and provide a defense against salinity stress in different plants [14].

## 2. Insight Mechanisms of Plants to Tolerate Salinity Stress

### 2.1. The Role of Osmolytes in Salt Tolerance

One of the main causes of the depolarization of the cell membranes, and cellular and metabolic system disorders that eventually harm the growth and yield of plants through disruptions in mRNA in protein synthesis (transcription and translation) and the inhibition of the activities of different enzymes, is osmotic stress, which is triggered by high salinity.

Plants modify their biochemical, subcellular, and metabolic processes in order to produce low molecular weight in physiochemically neutral small substances. They (Plants) do this to uphold cellular osmotic homeostasis by decreasing the Osmo-potential of stressed cells. The previously mentioned substances are concentrated in the cytosol of salt-stressed plants and are known as compliant solutes (osmoprotectants or osmolytes). They are non-toxic and have no negative effects on the cellular metabolic system. Osmoprotectants are typically found in the types of amino acid proline and sugar sucrose (e.g., wheat and pepper) glycine betaine (GB) (Trimethyl-glycine) (e.g., wheat), choline (e.g., chickpea) and polyamines (PAs), such as putrescine (e.g., tomato and *Cucumis sativus*), spermine (e.g., Arabidopsis, transgenic rice, tomato, and *Cucumis sativus*), spermidine (e.g., *Gladiolus gandavensis* and tomato), and sometimes the osmoprotectants may be found in the form of sugar alcohols, such as mannitol and sorbitol (e.g., rice) [15]. The mode of action of salt tolerance used by plants can be summarized in the following points:

1. Some plants are able to maintain high water potential by reducing the transpiration rate.
2. Salts are accumulated in stem and older leaves in which metabolic processes take place at a slower rate, so the plants target losing them.
3. Na^+^ toxicity is avoided by a backlog of a high amount of K^+^ ions.
4. Assemblage of toxic ions in the vacuole but not in the cytoplasm.
5. Accumulation of proline and abscisic acid.

#### How Ion Transporters Can Mediate the Tolerance of Salt Stress

In plants, the NHX1 protein-coding gene that is found in the tonoplast (the semipermeable membrane surrounding the vacuole) and plasma membrane-localized (integral) salt overly sensitive 1 (SOS1) protein, which is considered the leading and major protein to pump the excessive amounts of Na^+^ out of the cell, and is also recognized as a Na^+^/H^+^ antiporter (exchanger or counter-transporter). Both of them play a vital role in achieving ion homeostasis in plant cells.

Numerous enzymes in the cytosol are activated by K^+^ and inhibited by Na^+^ [16]. Plant cells can develop three modes of action to prevent an excessive buildup of Na^+^ in the compartment that is surrounded by the cell membrane (cytoplasm) [4]. First, according to the selective permeability property (i.e., letting some ions and molecules in and keeping some of them out), maybe selective ion absorption can limit the entry of Na^+^ into plant cells [17]. Second, vacuoles can hold absorbed Na^+^. Because the stored Na^+^ aids in osmotic regulation, vacuolar compartmentalization is a successful tactic for plant cells to oppose salt stress [15]. Third, Na^+^ is in the fluid present in the cell membrane and contains water, water-soluble proteins, and ions (cytosol) that may be transported back to the growth medium or to apoplastic areas that comprise the intercellular space filled with gases and H_2_O, including between cell membranes (i.e., the space between fibers and micelles of the cell walls). Na^+^/H^+^ counter-transporters on the plasma membrane are supposed to provide this function [18]. Na^+^ removal through these Na^+^/H^+^ exchangers is handled by an internal proton gradient triggered by the vacuolar proton-ATPase.

### 2.2. Hormonal Regulations in Salt-Stressed Plants

Small compounds called phytohormones, commonly referred to as plant hormones, are essential for the growth and development of plants. Salicylic acid (SA), brassinosteroids (BRs), strigolactones, and jasmonic acid (JA) are among the most common well-studied phytohormones that are classed as stress response hormones or hormone-like compounds. On the other side, the hormones auxins (e.g., IAA), gibberellin (GA), cytokinins (CTKs), abscisic acid (ABA), and ethylene are responsible for improving the growth of the plants, so they are regularly known as regulatory hormones or plant-growth hormones [19]. Many studies have demonstrated that each phytohormone has multiple bio-functions in plants. These functions are complex and effective depending on the stage of development, the tissue involved in the biological process, or the physical conditions of the surrounding environments [20,21,22,23].

#### 2.2.1. Osmosity Is Regulated by Abscisic Acid (ABA) in the Case of Salt-Stressed Plants

Abscisic acid is a plant hormone responsible for regulating plant growth and environmental stress responses, such as stimulating the closure of pores to prevent water loss. Structurally, the molecule of abscisic acid contains 15 carbon atoms and its formula is C_15_H_20_O_4_ [24]. Abscisic acid is called a stress hormone because it induces different reactions in plants against stress that increase plants’ tolerance to different stresses.

In the defense against salt stress, abscisic acid (ABA), which is one of the most significant stress response hormones, is critical [24]. ABA serves as a key that connects and rewires plants’ responsive signal cascades in responding to salt stress, particularly osmo-stress [25]. Endogenous abscisic acid levels rise quickly in response to salt and osmo-stress, and improved ABA signal initiates SnRKs (sucrose nonfermenting related protein kinases) [26].

SnRKs phosphorylate various abscisic acid-responsive element (ABRE)-binding protein/abscisic acid-responsive element (ABRE)-binding factor (AREB/ABF) transcription factors, which further manage stomatal closure in plants as feedback to osmo-stress [27]. Through the occurrence of salt stress, abscisic acid (ABA)-activated sucrose nonfermenting related protein kinases (SnRKs) also regulate osmotic equilibrium by controlling the two redox-sensitive enzymes included in starch destruction; the β-amylase1 (BAM1) and α-amylase3 (AMY3)-dependent breakdown of sugar energy storage (starch) into mono-sugars and sugar-derived osmoprotectants [28]. Abscisic acid (ABA) causes stomata (The major place where transpiration occurs) closure by impeding energy-dependent proton pumping into the plasma membrane of the guard cells, resulting in an influx of potassium, the relaxation of the guard cells, and the closure of the stomata in a process called “hydroactive stomatal closure”. ATP BINDING CASSETTE G25 (ABCG25) (an ABA exporter) and ATP BINDING CASSETTE G40 (ABCG40) (an ABA importer) cooperate to transport ABA to guard cells, which is a very important tactic for plants to handle NaCl-induced osmotic stress.

#### 2.2.2. Auxin-Mediated Root Growth Elasticity during Exposure to Salt Stress

The meristems absorb large amounts of water, which affects the growth of the apical tissues. This process is determined by a phenomenon called “growth in an acidic medium”, which explains the activity of auxins. It occurs when the polysaccharides and pectin that make up the cell wall soften due to the acidification of the medium. Cellulose, hemicellulose, and pectin lose their hardness, making it easier for water to enter the cell.

The root system of the plants displays growth elasticity regarding soil conditions, as auxin plays a critical role. There are three recognized categories of auxin receptors: AUXIN BINDING PROTEIN1 (ABP1) [29], S-PHASE KINASE-ASSOCIATED PROTEIN 2A (SKP2A) [30], and the nuclear SCF-TIR1/AFBs-Aux/IAA (SKP-Cullin-F box [SCF], TIR1/AFB [TRANSPORT INHIBITOR RESISTANT1/AUXIN SIGNALING F-BOX], AUXIN/INDOLE ACETIC ACID) auxin coreceptors.

Auxin is commonly sensed by the receptor TRANSPORT INHIBITOR RESPONSE 1 (TIR1) and the closely related AUXIN SIGNALLING F-BOX (AFB) proteins, which engage Aux/IAA repressors to the SCF-TIR1/AFB complex for ubiquitination (ubiquitylation or ubiquitinylation) which means adding of ubiquitin (small regulatory protein) to a substrate protein. It has different influences on proteins; it can enhance their degradation via the proteasome, modify their cellular localization, affect their activity, stimulate or prevent protein reactions, and ultimately activate auxin-induced gene expression [31]. By reducing auxin accumulation and suppressing the production of auxin receptors, salt stress also lowered the expression of the auxin receptor-encoding genes TIR1 and AFB2 [32], demonstrating that low auxin signaling is maintained to control plant growth adaptability.

#### 2.2.3. Plants Adjust Gibberellic Acid (GA) Levels to Cope with Salt Stress

The growth and propagation of plants on saline land are depending on the fruitful germination of the planted seeds, which is tightly related to both abscisic acid (ABA) and gibberellic acid (GA). The concentrations of GA in plants under salt stress change remarkably, demonstrating that GA and ABA may be intimately connected in the responses to abiotic stresses in plants [33].

Gibberellic acid (GA) links to the receptor GIBBERELLIN INSENSITIVE DWARF1 (GID1), which enhances a conforming change of GID1 and employs the growth-suppressing DELLA (aspartic acid–glutamic acid–leucine–leucine–alanine) proteins, which are a group of the GRAS (GIBBERELIC ACID INSENSITIVE REPRESSOR) DELLA, they are negative regulators of gibberellic acid (GA) signaling that behave immediately in a downward direction of the GA receptor. The binding of GA to its receptor GID1 causes the binding of GID1–GA to DELLAs and leads to the formation of a GA–GID1–DELLA complex, which stimulates their degradation via the ubiquitin–proteasome pathway.

#### 2.2.4. Plants Are Able to Withstand Salt Stress Thanks to Cytokinins CTKs Signal’s Altruism

Plants need cytokinins (CTKs) for a wide range of physiological and metabolic functions, such as cellular division, shoot:root ratios, progress and improvement, leaf senescence, and stress tolerance [34]. In addition, CTKs restrict the breakdown of proteins, chlorophyll, nucleic acids, and other chemicals in plants while dispersing essential amino acids, hormones, mineral salts, and other substances to various regions of the plant [35]. The messenger for the interaction between roots and shoots in the event of salt stress is suggested to be the transfer of CTKs from roots to shoots [36].

CTKs are viewed by the receptor Arabidopsis histidine kinase (AHK2/3/4)—which is localized on the membrane—and phosphorylated AHKs consequently transfer P groups to the type-B Arabidopsis response regulators (ARRs). ARRs are transcription factors (which are localized in the nucleus) through Arabidopsis histidine phosphotransfer proteins (AHPs), which play a significant role in cytokinin signaling through connecting the sensation of CTKs, by plasma-membrane receptors, to the triggering of cytokinin-responsive transcription factors [23].

#### 2.2.5. Responses of Plants to Salt Are Mediated by Jasmonic Acid (JA) and Comprising of Abscisic Acid (ABA)

Plant hormones work together to influence plant physiological interactions, growth, and improvement. Promising data indicates that jasmonic acid (JA) interacts with abscisic acid (ABA) to affect the plant response and tolerance to abiotic challenging conditions. It was mentioned above that the ABA can stimulate the plant’s response to abiotic stresses, such as salt stress. For instance, the expression of ABA biosynthesis genes expands in reaction to JA, and on the other hand, the expression of JA biosynthetic genes improves as a result of ABA synthesis.

The most important role of jasmonic acid (JA) was endorsed by many studies proving that the demonstration of JA biosynthetic and signal genes speedily and proactively dealt with biotic and abiotic stresses and that tolerance is impacted by the overexpression of the transcription factors NAC, MYB, and WRKY that respond to JA [37]. In response to salt, JA-promoted genes seemed to be expressed inside the inner tissue layer of roots, revealing that JA signaling is spatially controlled. As a result, there seem to be numerous possible targets for modifying abiotic stress tolerance in JA-related cascades.

## 3. Harmony between Synthesized ZnO-NPs, Defensive Antioxidants, and Osmoprotectants

Due to their unique physiochemical properties in the biological system, the utilization of nanoparticles (NPs) presents a chance for sustainability. Plant extracts serve as a source for the biological production—in each step of the synthesis—of many metallic nanoparticles that are manufactured with well-defined sizes and forms [38]. Metallic nanoparticles can also be synthesized using some of the chemical substances to produce chemical/or functionalized-chemical nanoparticles. Zinc oxide nanoparticles (ZnO-NPs), which are among all recognized metallic NPs, have a variety of benefits and uses since their synthesis is straightforward, affordable, workable, and utilizes less-toxic chemicals [39]. According to the molecular biology of abiotic stress, many field trials or in vitro experiments proved that the application of ZnO-NPs could increase growth attributes, such as the germination and yield of some crops, including *Oryza sativa* and *Hordeum vulgar* [40,41]. Zhang et al. showed that—by applying ZnO-NPs—the *O. sativa* yield was enhanced and ranged from 2.5% to 11.8%. These findings indicated that ZnO-NPs have a positive impact on plants, such as rice, under normal conditions at low-dose additions. So, if they (ZnO-NPS) produce this promising effect on plants under normal conditions, it is surely supposed to have the same effect on the same kinds of plants under stress.

There are many plants that adaptively tolerate salt stress with the help of ZnO-NPs as shown in Table 1, such as cotton (*Gossypium barbadense* L.) [42], sorghum (*Sorghum bicolor*) [43], tomato (variety PKM-1) [13], soybean (cv. Giza111) [44], soybean (*Glycine max* L.) [14], safflower (*Carthamus tinctorius* L.), rapeseed (Okapi cultivar and *Brassica napus* L.), *Triticum aestivum* L. (Inqilab 91 and Pasban 90), *Salvia officinalis*, *Zea mays*, *Abelmoschus esculentus* L. Moench, spinach (*Spinacia oleracea* L.), and lupine (*Lupinus termis*) [45,46,47,48,49,50,51,52,53].

In soybean, the exhibition of soybean (cv. Giza111) seedlings to salt stress in a concentration of 0.25 M NaCl showed unfavorable effects on germination % and plant growth features that are induced and are known to result from water shortages, osmotic stress, and metabolic abnormalities [44]. Since zinc element (Zn) is considered a vital mineral for plant growth and development [54], previous work found that soaking soybean seeds in ZnO-NPs—that were synthesized using the completely chemical bath deposition (CBD) method—before sowing them boosted the germination rate and other growth features in the seedlings of NaCl-stressed plants. Zinc element is crucial for the synthesis of natural auxin IAA, as it stimulates the biosynthesis of amino acid tryptophan, which is considered the precursor of indole-based auxins. Indole-based auxins are responsible for the longitudinal growth of plants that affect cell elongation. Auxins can also scavenge the free radicals, transfer nutrients from elderly cells to new ones (leading to the reduction of the uptake of the excessive amount of Na^+^), stimulate cell division, provide the preservation of the cell membrane, accumulate the functional phospholipids, and play a critical role in the protein synthesis process [55].

Soliman et al. proved that seedlings previously soaked in 50 mg/L of ZnO-NPs showed the fullest improvement in growth attributes under normal and saline treatments, indicating an excellent enhancing effect of such concentration on salt-stressed or non-stressed seedlings. It was hypothesized that adding ZnO-NPs to plant seeds with increased Zn concentrations would raise germination rates and other growth characteristics [52]. It may also occur because ZnO-NPs have the potential to boost the optimum size and turgidity pressures by enhancing water usage effectiveness and increasing moisture content. The ZnO-NPs may also decrease the toxicity of Na^+^ by reducing the absorption level of the plant to this ion, which leads to scavenging the osmo-potential [56].

In a study of sorghum (*Sorghum bicolor*), ZnO-NPs were synthesized using a completely green synthesis protocol with *Agathosma betulina* extract, and the concentration of the stressor (NaCl) was 0.4 M [43]. That extract was considered the reductant, the capping (stabilizing) agent, and the precursor for the metallic encounter zinc nitrate (ZnNO_3_). The ratio of macronutrients was also examined in order to comprehend the relationship between the damaged morphological features and the growth restriction brought on by osmotic stress. In the previous study, it was found that there is a relation between the rise in toxic ions, such as Na^+^ and Cl^−^, and a reduction in the immersion of essential elements that are playing a crucial role in the growth, and this was related to the high Na^+^/K^+^ ratio of 2.9 for treated plants. On the other side, after the priming of salt-stressed sorghum plants using the synthesized ZnO-NPs, the ratio of Na^+^/K^+^ was decreased to 1.53 (in the case of the concentration of 5 mg/L of ZnO-NPs), and the lowest ratio was 0.85 (in the case of the concentration of 10 mg/L of ZnO-NPs).

This is considered true, as salt stress damages the cell membrane due to the oxidative stress on the compartments of the membrane. So, by priming with chemically synthesized ZnO-NPs, these effects were overturned. Abiotic stressors typically result in a significant concentration of free radicals in plants, which stimulates the activation of antioxidants to maintain equilibrium and minimize lipid peroxidation [57]. So, the previous study also aimed to assess the degree of oxidative damage in salt-stressed plants by detecting reactive oxygen substances/species (ROS) accumulation, the peroxidation of lipids, and the impairment of biomolecules.

Reactive oxygen substances (ROS)—hydroxyl ion OH^−^, superoxide anion O^.^_2_^−^, and hydrogen peroxide H_2_O_2_—are signal molecules at biochemical levels, but their increased production destroys biological molecules (proteins, lipids, nucleic acids, etc.) and causes membrane oxidative damage through elevating lipid peroxidation [58]. In accordance with their morphological traits, salt-treated sorghum plants cumulated large levels of ROS and Malonaldehyde (MDA) (a biomarker of oxidative stress in plants; one of the final products of polyunsaturated fatty acids peroxidation in the cells). The secondary oxidative stress that occurs as a result of primary abiotic stress damages the lipid bilayer of cell membranes. The level of malondialdehyde, which is formed from the compounds derived from lipid peroxidation, increases as a result of oxidative stress, which can be used as a biomarker. On the other side, sorghum plants that were primed with ZnO-NPs showed low levels of these oxidative stress biomarkers. FTIR was used to investigate any alterations in the spectral peaks of the sorghum shoots in order to detect any damage to the biomolecules, such as proteins (responsible for functions), carbohydrates (responsible for molecular recognition), or lipids (considered the matrix of the cells). The Fourier-transform infrared spectroscopy (FTIR) spectra (as a confirmatory analytical device) showed shifts in the spectral peaks corresponding to carbohydrates, indicating that biomolecules in salt-treated sorghum plants were destroyed (2916.39 and 1637.12 cm^−1^) and proteins (1250.84 and 582.11 cm^−1^) were discovered. On the other side, peaks from the 10 mg/L ZnO-NPs-primed plants were more similar to the non-stressed plants (control), indicating that the priming of seeds of sorghum plants with ZnO-NPs improved sorghum’s sensitivity to salt stress and prevented the destruction of the mentioned biomolecules.

In another experiment, Faizan et al. (2021) used tomato seeds from the PKM-1 variety to apply the foliar treating of ZnO-NPs, which were obtained from Sigma-Aldrich, Inc. Chemicals Pvt. Ltd., Missouri, St. Louis, MO 68178, United States of America (USA), as regulators to improve growth parameters in plants under NaCl stress (150 mM), as it is thought—as mentioned in many different studies—that Zn element is essential for the production of proteins, Aux/indole acetic acid, and chlorophyll [13]. It also protects the configurational structures (structural stability) of biological membranes and inhibits the uptake of extra Na^+^ and Cl^−^ [59]. Furthermore, Zn is also an important cofactor of many enzymes, such as superoxide dismutase (SOD) and glutathione dehydrogenase (GDH) [60]. The partitioning of the superoxide (O_2_^•^) radical is created as a result of the transformation of biochemically used oxygen into molecular oxygen (O_2_), and H_2_O_2_ is mediated by the enzyme SOD in an alternative manner [61,62]. Although glutathione’s intricate and detoxifying properties have long been understood [63,64], a recent study has also uncovered unique aspects [65]. The ability of GST (glutathione transferase) genes to be activated by a variety of stressors is a distinguishing trait of these genes. Different classes of GSTs are selectively activated in the early stages of the infections by bacteria, fungi, or viruses according to numerous transcriptome studies. Numerous investigations showed that the rate of pathogen propagation, and consequently, the severity of the stream of infections, are strongly influenced by the up- or downregulation of GSTs [65].

The findings were in line with other researchers’ investigations of several plants, including green peas, moringa, and onion [66]. Stomatal closure caused by the presence of increased quantities of NaCl lowers the osmotic potential [67], and also the net photosynthetic rate (PN), intercellular CO_2_ (Ci) concentration, and stomatal conductance (Gs).

The results of the study showed that, in comparison to plants treated with NaCl alone, plants treated with ZnO-NPs in the occupancy or lack of NaCl performed much better in terms of PN, gs, and Ci, as well as chlorophyll content. The plants exposed to 50 mg/L of ZnO-NPs showed the greatest improvement. By accelerating the splitting of the water molecule and the exchange of electrons through redox processes, NPs improved photosynthesis and other associated properties [68].

The activity of Ribulose 1,5-bisphosphate carboxylase/oxygenase enzyme (Rubisco) was upgraded by using ZnO-NPs, which was directly associated with increased photosynthetic apparatus activity [69]. In the previous study, tomato seedlings cultivated in soil that contained sodium chlorite had lower protein content. NaCl-induced veracity of the cellular coverage, coupled with cellular machinery that contains proteins and excessive ROS production, are the causes of this diminution [70]. On the other hand, it was discovered that there was an increase in the percentage of protein proportion following the treatment of salt-stressed tomato seedlings with ZnO-NPs (10, 50, or 100 mg/L), which proved the beneficial impacts of ZnO-NPs in the exacerbation of salt stress. The increased protein concentration following Zn administration may be related to the reduced ion leakage, which lessens the damage caused by NaCl stress [71]. It is very important to prevent oxidative damage; it is crucial to keep a balance between the generation and breakdown of ROS. Therefore, ROS-scavenging antioxidant enzymes, such as SOD, peroxidase (POX/APX), and catalase (CAT), play a critical role in detoxifying ROS in plants [72]. APX and CAT have the capability to neutralize the cumulative hydrogen peroxide (H_2_O_2_) by converting it into H_2_O [73].

One significant finding of that research was that ZnO-NPs applied topically improved antioxidative enzyme activity in both the presence and absence of NaCl as an abiotic stressor. Previous studies have shown that ZnO-NPs boosted the antioxidant defense system and increased the activity of SOD, POX, and CAT, which was a response to the transcription of genes or their involvement in numerous oxidative processes [74,75,76].

## 4. Synthesized ZnO-NPs and Molecular Parameters in Salt-Stressed Plants

By identifying and attaching to common regions in the operator of downregulation involved in stress reactions, transcription factors (TFs), the terminal transducers in a signaling cascade, can control the expression of these genes.

The effect of synthesized zinc oxide nanoparticles on the transcriptional factors of salt-stressed plants was tested using rapeseed (*Brassica napus* L.) [47]. In that study, three concentrations of NaCl were used: 0, 50, and 100 mM. ZnO-NPs were purchased and suspended in de-ionized water, dispersed using ultrasonics, and applied in three different concentrations: 0, 20, and 80 mg/L. The gene expression profile of the auxin-responsive protein (ARP) was created in response to the treatment of non-stressed and stressed *Brassica* using ZnO-NPs that were foliar sprayed. It was found that the ARP gene expression was reduced once the salinity increased. In another study, it was proven that, in the case of the exposure of wheat plants to NaCl stress, the total amount of auxins will be decreased [77]. It is thought that salinity stimulates the production of reactive oxygen species (ROS), which then activate auxin oxidases enzymes; therefore, the activities of numerous genes involved in growth processes are expected to be reduced as a result of enhanced auxin breakdown caused by auxin oxidases.

For myelocytomatosis oncogenes (MYC) transcription factors—which are key TFs responsible for the expression of jasmonic acid-responsive genes and are included in the biosynthesis of anthocyanins, some amino acids, such as tryptophan (Tryp), stomatal differentiation, seed germination, and endosperm breakdown—it was noticed that the plants that had been treated with ZnO-NPs (20 mg/L) had the highest expression of MYC. The expression of MYC showed a decreasing trend under salinity. The degradation of the precursors of MYC may be responsible for the decrease in their level during salinity [77]. The increase of the reactive oxygen species, such as H_2_O_2_, may also be responsible for that decrease, which is followed by DNA damage.

Under conditions of salt stress, the mitogen-activated protein kinases (MAPKs or MPKs) pathway that controls the generation of antioxidants and osmoprotectants must be studied. MPK3 and MPK4 were identified in that study under co-treatments with salinity. The mRNA synthesis of MAPK3 (a part of the response to the modeling process of the stomata) decreased with increasing salinity concentration in rapeseed. The highest effect of ZnO-NPs in decreasing the expression of MAPK3 was observed under NaCl (100 mM). The MPK3 transcript was found to be decreased by the increase in the level of NaCl, and at the level of 100 mM of NaCl, the highest effect of ZnO-NPs was found. It might be connected to the MAPK product’s lifetime, which is dependent on upstream-specific regulatory mechanisms, the most known of which are construction (co-localization) and attenuation through phosphatases [78,79]. On the other hand, at the concentration of 100 mM of NaCl, it was found that MAPK 4 had the highest expression after the treatment of zinc oxide nanoparticles. There was a positive correlation between MAPK4 and the treatment of salt stress with ZnO-NPs. Therefore, it is clear that some MAPKs are activated or inactivated under salinity, so the regulation of MAPKs under salt stress improves the cascade of reactions that play an important role in enhancing the salt tolerance of the plants.

## 5. Conclusions

Due to the increasingly alarming problems that soils and plants are exposed to as a result of different types of abiotic stresses, such as salinity, drought, low temperature (chilling or frost stresses), or high temperature, more attention must be introduced to studying how plants can mitigate the negative effects of these stresses on them. For example, as is known for salinity stress, plants lose a very large amount of their vitality and ability to complete various biochemical processes, which affects the yield of the crops. Hence, it shows the importance of studying the various factors that regulate the variant processes of plants under these conditions of stress, such as osmoprotectants and phytohormones. Further, the exploitation of some nanoparticles, such as ZnO-NPs, that have shown promising results in alleviating salinity stress—whether by their dealing with different transcription factors or even by stimulating some hormones and compounds that regulate osmotic pressure inside the plant cell—helps maintain the protein’s integrity, selective permeability, photosynthetic rate, and other vital processes.

## Figures and Tables

**Table 1:** Examples of some plants that are adaptively-salt stress developed by ZnO-NPs.

Nanomaterial	Plant	Conc. of NaCl	Reference
**Zinc oxide nanoparticles (ZnO-NPs)**	Cotton (*Gossypium barbadense* L.)	10% and 20% seawater	[42]
	Sorghum (*Sorghum bicolor*)	0.4 M	[43]
	Tomato (variety PKM-1)	150 mM	[13]
	Soybean (cv. Giza111)	0.25 M	[44]
	Soybean (*Glycine max* L.)	250 mM	[14]
	Safflower (*Carthamus tinctorius* L.)	250 mM	[45]
	Rapeseed (Okapi cultivar)	50 and 100 mM	[46]
	Rapeseed (*Brassica napus* L.)	0, 50, and 100 mM	[47]
	*Triticum aestivum* L. (Inqilab 91 and Pasban 90)	Saline water (EC = 6.3 dS m^−1^)	[48]
	*Salvia officinalis*	75, 100, and 150 mM	[49]
	*Zea mays*	75 and 150 mM	[50]
	*Abelmoschus esculentus* L. Moench	10, 25, 50, 75, and 100% seawater	[51]
	Spinach (*Spinacia oleracea* L.)	100 mM	[52]
	Lupine (*Lupinus termis*)	150 mM	[53]

## Data Availability

Not applicable.
